# Scintillation Properties of CsPbBr_3_ Quantum Dot Film-Enhanced Ga:ZnO Wafer and Its Applications

**DOI:** 10.3390/ma18153691

**Published:** 2025-08-06

**Authors:** Shiyi He, Silong Zhang, Liang Chen, Yang Li, Fangbao Wang, Nan Zhang, Naizhe Zhao, Xiaoping Ouyang

**Affiliations:** 1 National Key Laboratory of Intense Pulsed Radiation Simulation and Effect, Northwest Institute of Nuclear Technology, Xi’an 710024, China; hesyth10@sina.com (S.H.); 18811617658@163.com (Y.L.); wfb0619@163.com (F.W.); 18264861969@163.com (N.Z.); zhaonaizhe@nint.ac.cn (N.Z.); ouyangxiaoping@nint.ac.cn (X.O.); 2 School of Materials Science and Engineering, Xiangtan University, Xiangtan 411100, China; tay552296@163.com

**Keywords:** Ga:ZnO, perovskite quantum dots, cryogenic properties, radioluminescence, pulsed radiation detection

## Abstract

In high energy density physics, the demand for precise detection of nanosecond-level fast physical processes is high. Ga:ZnO (GZO), GaN, and other fast scintillators are widely used in pulsed signal detection. However, many of them, especially wide-bandgap materials, still face issues of low luminous intensity and significant self-absorption. Therefore, an enhanced method was proposed to tune the wavelength of materials via coating perovskite quantum dot (QD) films. Three-layer samples based on GZO were primarily investigated and characterized. Radioluminescence (RL) spectra from each face of the samples, as well as their decay times, were obtained. Lower temperatures further enhanced the luminous intensity of the samples. Its overall luminous intensity increased by 2.7 times at 60 K compared to room temperature. The changes in the RL processes caused by perovskite QD and low temperatures were discussed using the light tuning and transporting model. In addition, an experiment under a pico-second electron beam was conducted to verify their pulse response and decay time. Accordingly, the samples were successfully applied in beam state monitoring of nanosecond pulsed proton beams, which indicates that GZO wafer coating with perovskite QD films has broad application prospects in pulsed radiation detection.

## 1. Introduction

Through the development of inertial confinement fusion, laser–matter interaction, and many plasma physics experimental studies [1,2,3], many new uncertain fast physical processes have occurred under extreme conditions, which require the advanced use of fast pulsed radiation detection methods to understand the mechanisms and observe the processes [4,5,6]. Detection systems centered on ultra-fast scintillators are among the most important systems for fast-pulsed radiation detection and play key roles in the fields of nuclear medicine, environmental monitoring, and space exploration [7,8,9]. New scintillators with nanosecond or shorter decay times and luminous intensities of tens of thousands or higher have always been a key direction and target in the field of materials [10,11,12].

However, most current materials, including SrI_2_(Eu) [13], BaF_2_ [14], quenched plastic scintillators [15], wide-band materials [16], and perovskites [17], are unable to achieve a balance between the two. This indicates that achieving both a short decay time and high efficiency through a single material is very difficult, whether organic or inorganic. Metamaterials provide an alternative approach to enhance the performance of scintillating materials [18]. Artificial microstructures can be attached to scintillating materials to regulate specific luminescent properties [19,20,21]. In addition to traditional structural composite methods, a large amount of research has been conducted to enhance detection efficiency, luminous intensity, angular distribution, decay time, and other properties of scintillating materials via photonic crystals, photon-structured nanospheres, quantum wells, annealing, nanoimprinting, and others [22,23,24].

The radioluminescence (RL) of Ga:ZnO (GZO) was successfully enhanced in previous studies by the wavelength tuning effect of a CsPbBr_3_ quantum dot (QD) film coated on the surface of GZO [25]. This method overcame the problems of self-limited luminescence and self-absorption in wide-bandwidth materials, enhanced their luminous intensity and optical path, and addressed their luminescence and thickness limitations [26].

In addition to the enhancement of RL, the use of perovskite QD films as metascintillators on base materials such as GZO has more benefits. The external quantum efficiency will be increased because the refractive index of the film lies between that of GZO and air; the long-term stability of QD will be improved since GZO is dense enough to isolate air and moisture; and the easier processability of QD films compared to the hard and brittle GZO wafers was more conducive to fabricating various microstructures [27]. The 380 nm emission of ZnO perfectly covers the exciton absorption band of CsPbBr_3_ in the UV region; therefore, CsPbBr_3_ is a suitable choice. In addition, since perovskite materials are now diverse and energetic, discovering faster perovskite QD with similar luminous intensity and transform efficiency is greatly promising [28,29,30].

This study primarily investigated a three-layer-type sample based on GZO. It analyzed changes caused by CsPbBr_3_ QD films in RL spectra and decay times based on characterization results. The enhancement of RL and the intensity ratio between GZO and CsPbBr_3_ QD films were obtained. Results for each surface were compared and comprehensively discussed using the light tuning and transport model. In addition, an experiment using a pico-second electron beam was conducted. As an application, data from a nanosecond pulsed proton beam detected by the samples were presented, verifying the samples’ broad potentials in the field of pulsed radiation detection.

## 2. Materials and Methods

GZO single crystals were fabricated by Ganjiang Innovation Academy, Chinese Academy of Sciences, Ganzhou, China. A hydrothermal method was utilized to grow high-quality gallium-doped ZnO bulk single crystals [31,32]. The thickness of the GZO single crystals was 0.20 ± 0.02 mm, and the carrier concentration was ~1 × 10^19^ cm^−3^. A CsPbBr_3_-dodecyl benzene sulfonic acid QD solution was sprayed onto the surfaces of GZO wafers at a constant speed to form QD films. A step profiler (Dektak XT, Bruker, Berlin, Germany) was utilized to measure the thickness of the QD films.

The RL spectra of GZO and the composite samples were measured. An X-ray generator (12 WX-ray source, Moxtek, Orem, UT, USA), a photomultiplier tube (PMT, 77360, Newport, Irvine, CA, USA), a spectrometer (74126, Newport, Irvine, CA, USA), and a power meter (1936-R, Newport, Irvine, CA, USA) were used.

A homemade time-correlated single-photon counting (TCSPC) system was utilized to acquire the decay time of the samples. The system mainly included a PMT (9815, ET Enterprises Limited, London, UK) for signal acquisition, a microchannel plate (MCP, R3809U-50, Hamamatsu, Hamamatsu City, Japan) for single proton signals, and a series of electronic devices. A neptunium (Np) source producing 4.7 MeV alpha particles was utilized to excite the samples.

The impulse response of the scintillator was measured using a pico-second pulsed electron linear accelerator at the Northwest Institute of Nuclear Technology. The accelerator uses an ultrashort ultraviolet-driven laser to irradiate a photocathode, generating electrons, which form a pulse cluster at the outlet through two long traveling wave accelerator tubes. A single pulse contains 500 pC of charge, and its pulse width is about 10 ps.

Pulsed proton beam monitoring was conducted using a laser-pulsed proton source at the Shanghai Institute of Optics and Fine Mechanics. Protons were generated by irradiating a target with a high-energy laser. Then, protons were deflected, collected, and compressed by a magnet to form a pulsed proton beam. The particle count of each pulse was about 10^7^ at maximum, and the pulse width ranged from 2 to 4 ns. Due to the changing magnetic field, the intensity and width varied continuously. The pulse beam status and control parameters did not exhibit a simple one-to-one correspondence and required special monitoring.

## 3. Results and Discussion

**Figure 1:**
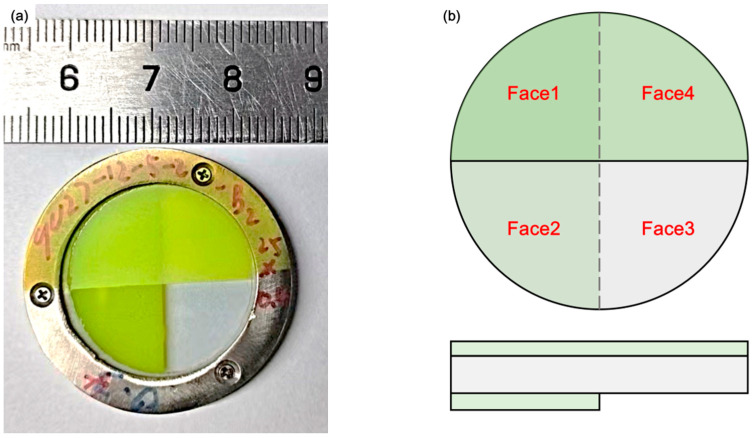

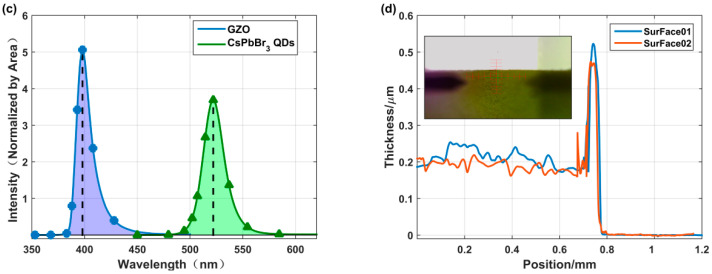
Structure and characterization: (**a**) photos of a three-layer sample; (**b**) multilayer structure of the sample and faces definition. The sample structure of Face1, Face2, Face3, and Face4 are CsPbBr3/GZO/CsPbBr3, GZO/CsPbBr3, GZO, and CsPbBr3/GZO; (**c**) RL spectrum of GZO and PL spectrum of CsPbBr_3_ QD; (**d**) thickness and flatness of CsPbBr_3_ QD films on each surface.

A typical scintillator sample was shown in Figure 1a, consisting of a 0.2 mm GZO wafer in the middle and two CsPbBr_3_ QD films attached to its surfaces. The structure of the sample was illustrated in Figure 1b. For convenience, four areas were marked on the emitting surface as Face1 through Face4. The RL spectra of the GZO crystal and CsPbBr_3_ QD were measured and shown in Figure 1c. The luminous spectra of the two materials were acquired, with peaks located at 398 and 522 nm, respectively. The CsPbBr_3_ QD films in Figure 1d significantly changed the surface morphology of the GZO wafer. The thickness of the CsPbBr_3_ QD films was about 210 nm using a step profiler.

The process of the sample interacting with radiation followed a cascade of ‘radiation-light-modulated light’. Since the CsPbBr_3_ QD films were much thinner than the GZO wafer, most of the radiation energy was deposited in the GZO. The ultraviolet light (~398 nm) emitted by the GZO fell within the absorption spectrum of the CsPbBr_3_ QD and was easily absorbed by the tens-of-nanometers-thick CsPbBr_3_ QD films in large proportions. Then, the CsPbBr_3_ QD transitioned from the excited state to the ground state after vibrational relaxation, emitting light within the wavelength range of their photoluminescence (PL) spectrum. These secondary emissions were uniformly and spatially distributed and lay beyond the absorption spectrum of the GZO, resulting in weaker loss and damping. As a result, the CsPbBr_3_ QD films tuned part of the light that would otherwise have been absorbed or emitted in other directions, enhancing the luminous intensity of the GZO wafer.

**Figure 2:**
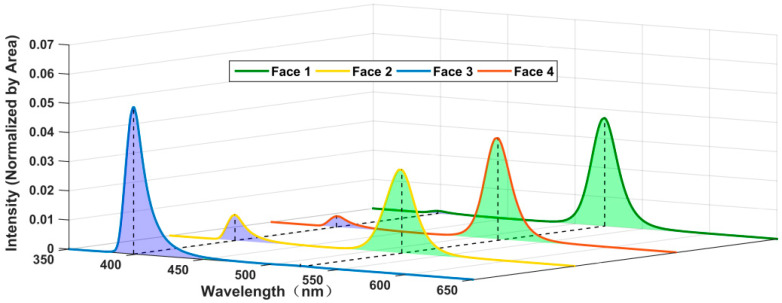
RL spectra from 4 different faces on emitting surface excited by X-ray.

The RL spectra of each face were shown in Figure 2. Each spectrum included two peaks at 398 and 522 nm but with differing proportions. Based on the spectra in Figure 1c, the former peak originated from the GZO and the latter from the CsPbBr_3_ QD. Fitting was performed on each spectrum in Figure 2, using the RL of the GZO and the PL of the CsPbBr_3_ QD. The area ratios of the two peaks (QD/GZO) for each face were 55.0, 5.0, 0.04, and 12.1, respectively. The tuning behavior of the CsPbBr_3_ QD on the GZO RL was significant, and the conversion efficiency increased with the layer and its thickness.

A tuning coefficient *a* was utilized to quantify the quantum efficiency during the process in which photons from the GZO RL passed through the CsPbBr_3_ QD films. A multiplication coefficient *k* was utilized to quantify the absorption and secondary luminescence of the CsPbBr_3_ QD, since the average energy of photons from the CsPbBr_3_ QD was less than that from the GZO. Assuming energy conservation, the upper limit of *k* was calculated as shown in Equation (1), based on the spectra in Figure 1c.

(1) $$k=\frac{\bar{E_{c}}}{\bar{E_{z}}}=\frac{\sum f_{c}\left(\lambda_{i}\right)\frac{1}{\lambda_{i}}\Delta \lambda_{i}}{\sum f_{z}\left(\lambda_{i}\right)\frac{1}{\lambda_{i}}\Delta \lambda_{i}}=1.30$$

where *k* = 1.30, *E* is the average energy, *f* is the spectral distribution, subscripts Z and C are the GZO and CsPbBr_3_ QD, *λ*_i_ is the wavelength at the center of each bin, and Δ*λ*_i_ is its bin width.

Based on the previous model developed by the research group [25], an equation set was established to obtain the tuning coefficient *a* and two optical transfer parameters (*m*_1_ and *m*_2_) from the area ratios (QDs/GZO) in the RL spectra of Face1, Face2, and Face4, as shown in Equation (2). *a* = 94.9%, indicating that 94.9% of the GZO RL entering the films was converted into CsPbBr_3_ QD PL.

(2) $$\left\{\begin{array}{l} \frac{\alpha km_{1}+\alpha km_{2}}{2m_{1}\left(1-\alpha \right)}=55.0\\ \alpha km_{2}/2=5.0\\ \frac{\alpha km_{1}/2}{m_{1}\left(1-\alpha \right)}=12.1 \end{array}\right.$$

**Figure 3:**
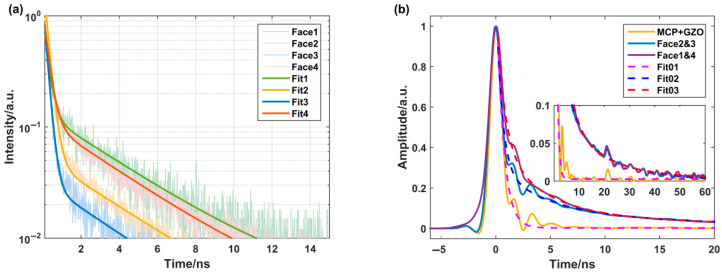
Temporal characteristics of the sample: (**a**) decay curves of different faces measured by TCSPC system excited by alpha particles; (**b**) pulsed waveforms excited by a pico-second pulsed electron linear accelerator.

The results of the sample’s decay time tests were illustrated in Figure 3a. The decay curve of all the samples can be fitted by the biexponential function. In addition to the components from the GZO, a 3.9 ns slower decay time component was observed in each curve, originating from the CsPbBr_3_ QD films. From highest to lowest, the proportions of the slower decay time component were Face1, Face4, Face2, and then Face3. This pattern was consistent with the RL spectra results shown in Figure 2.

**Figure 4:**
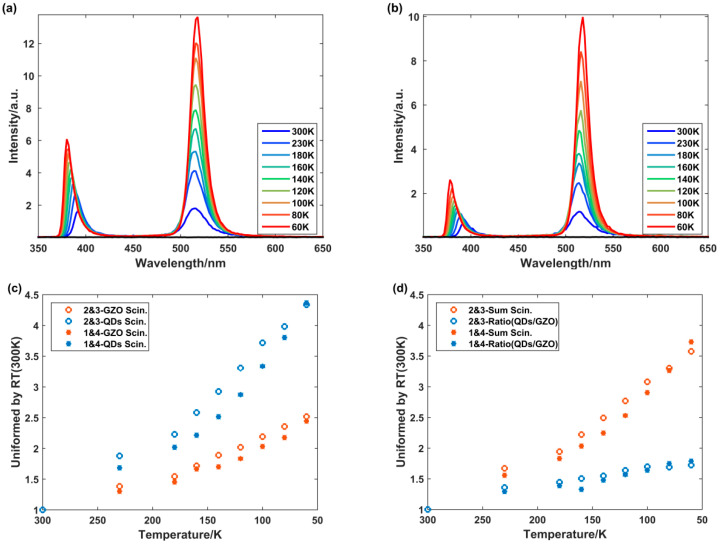
Cryogenic properties of cascaded sample: (**a**) sample’s RL spectra changing with temperature when GZO was the emitting surface (Face2 and Face3); (**b**) sample’s RL spectra changing with temperature when CsPbBr_3_ QD film was the emitting surface (Face1 and Face4); (**c**) variation of the area from different components with temperature; (**d**) variation of the total luminescence intensity and the area ratio (QDs/GZO) with temperature.

The pulse response of the cascaded samples was obtained utilizing a pico-second pulsed electron linear accelerator, as shown in Figure 3b. The half-height width, rise time, and fall time of the pulse were approximately 1.5, 1.1, and 7.2 ns, respectively. An obvious slower rear edge was observed, which was not present in the waveform obtained from the GZO wafer. This indicated that the tail originated from the CsPbBr_3_ QD films. Between the luminescence emitted from the GZO surface (Face2 and Face3) and the CsPbBr_3_ QD films (Faces1 and Face4), slight differences were observed in the tail, specifically in the proportion of CsPbBr_3_ QD luminescence. After fitting, three decay time components, 0.7, 4.2, and 18 ns, were obtained, which were slightly higher than the decay times measured by the TCSPC system excited by alpha particles in Figure 4a.

The variation of the sample’s RL spectrum excited by X-ray with temperature was obtained, as shown in Figure 4a,b. For each type of emitting surface (GZO or CsPbBr_3_ QD film), no conditions or layouts were changed during the experiments. Divided by the value at room temperature (300 K), variations of the area from GZO, the area from CsPbBr_3_ QD, the ratio of the two (QDs/GZO), and the total RL intensity with temperature were extracted from the spectra, as depicted in Figure 4c,d.

As the temperature decreased, the RL intensity of the two emission bands significantly increased. Given that both emission bands originate from excitonic transitions (free or bound excitons), the reduction in temperature effectively suppresses multiphonon-assisted non-radiative recombination (thermal quenching), thereby enhancing the radiative recombination efficiency and resulting in a significant increase in RL intensity [33]. At 60 K, the area from GZO was about 2.5 times that at RT, and the area of CsPbBr3 QD was 4.4 times that at RT. The overall luminescence intensity of the sample increased by 2.7 times, while the area ratio increased by 75%. As the temperature decreases, the GZO emission peak exhibits a blue shift, which can be attributed to the Varshni effect [34]. However, CsPbBr_3_ QD emission shows an unusual red shift, contrary to the phenomenon observed in conventional semiconductors. The emission peak position exhibits a nearly linear increase as the temperature decreases from 300 K to 60 K. As shown in Figure 4b, the red shift is considered to originate from the synergistic effect of electron–phonon coupling and lattice thermal expansion [35]. The differences in increment between the area of GZO and CsPbBr_3_ QD indicated an improvement in the CsPbBr_3_ QD absorption and secondary luminescence process, which was quantified as the tuning coefficient *a*.

Theoretically, it was known from Equation (2) that the area ratio of CsPbBr_3_ QD to GZO in each spectrum was proportional to *ka*/(1 − *a*). Thus, rises in the area ratio in Figure 4 (to 175%) were caused by both the multiplication coefficient *k* and the tuning coefficient *a*. The value of *k* at 60 K differed from that at 300 K due to the change in the RL spectrum. Based on Equation (1), *k* becomes 1.36 at 60 K. Therefore, Equation (3) indicated that *a* increases from 94.6% to 96.7% at 60 K. The cascaded sample showed a trend of RL enhancement at low temperatures under the combined effect of the RL intensity of the GZO and *a*.

(3) $$\frac{\alpha_{30K}k_{30K}}{\left(1-\alpha_{30K}\right)}/\frac{\alpha k}{\left(1-\alpha \right)}=1.75\Rightarrow \alpha_{30K}=96.7\%$$

In principle, the luminescence of both CsPbBr_3_ QD and GZO is edge-banded luminescence [36,37,38,39]. In edge luminescence, the absorption spectrum and the luminescence spectrum are highly coincident. Due to the influence of thermal motion and phonons, both the edges of the absorption spectrum and the emission spectrum exhibit wavelength (energy) broadening. Based on the Arrhenius equation, with decreasing temperature, the lattice energy of the material decreases, the extent to which phonons participate in luminescence weakens, and the broadening of the energy spectrum narrows. In addition, the overlap between the absorption spectrum and the luminescence spectrum decreases, macroscopically resulting in higher emission intensity.

The peak of the luminescence spectrum also changes slightly with temperature. Decreasing temperature can also lead to other property changes in the material, such as thermal motion and bandwidth, which also affect the luminescence spectrum and peak positions of the material. As the temperature decreases, the luminescence of GZO undergoes a blue shift, while that of CsPbBr_3_ QD exhibits a red shift. The influence of temperature on peak positions is not constant and varies among different materials.

**Figure 5:**
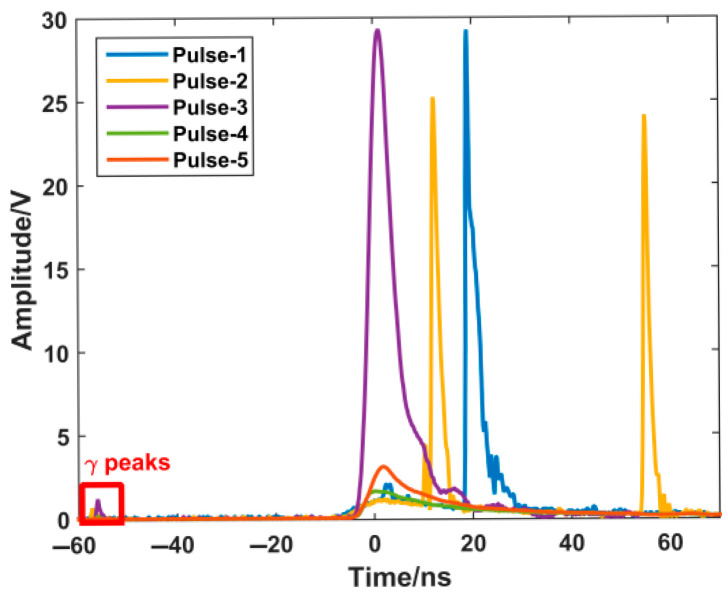
An application: typical results detected by samples in a nanosecond pulsed proton beam monitoring.

As an application in the field of pulsed radiation detection, this scintillator was successfully used in the status monitoring of nanosecond pulsed proton beams. In the measurements of pulsed electrons and protons, oscilloscopes (HDO8108A, Teledyne LeCroy, Chestnut Ridge, NY, USA) were used for data acquisition. Some typical statuses of the proton beam monitored by the samples were shown in Figure 5. GZO coated with CsPbBr_3_ QD films could distinguish various beam statuses caused by parameter changes or drift. In experiments, the scintillator assisted in identifying and locating nanosecond-wide low-intensity components. Since the temporal characteristics of the proton beam were slower, the test results of cascaded scintillators were beneficial for pulse recognition of the broadening and attenuation of proton quantity and energy. A gamma peak signal appeared in each waveform. Gamma was generated during the interaction of the laser and the target and transported along a straight line. Thus, the peak energy of the proton pulse, as well as its energy spectrum, could be obtained by samples using the time-of-flight method.

## 4. Conclusions

This study studied a cascaded scintillator combined with a GZO wafer and two CsPbBr_3_ QD films coated on both surfaces. Due to the presence of CsPbBr_3_ QD films, a new peak at 522 nm was generated in the RL spectrum, and two new decay time components of 4.2 and 18.6 ns were produced. With different layer structures, a significant difference was observed in the area ratio between the new CsPbBr_3_ QD peak and the original GZO peak. The proportion of CsPbBr_3_ QD increased with the increase in its layer quantity and thickness. At low temperatures, the luminescence intensity of the sample increased by 2.7 times, the area ratio of CsPbBr_3_ QD to GZO increased by 75%, and the luminescence of GZO and quantum dots was 2.5 times and 4.4 times that at room temperature, respectively. In addition, based on the optical model, the tuning coefficient of CsPbBr_3_ QD films increased from 94.6% to 96.7%. The cascaded sample was successfully applied to monitor a nanosecond pulsed proton beam. The results and applications above indicates that the samples have broad potential in the field of pulsed radiation detection.

## Data Availability

The original contributions presented in this study are included in the article. Further inquiries can be directed to the corresponding author.

## Abbreviations

The following abbreviations are used in this manuscript:

GZO	Ga:ZnO, gallium doped into zinc oxide
QD	Quantum dot
RL	Radio luminescence
PL	Photo luminescence
TCSPC	Time-correlated single-photon counting system
